# Ultra-Sensitive Photo-Induced Hydrogen Gas Sensor Based on Two-Dimensional CeO_2_-Pd-PDA/rGO Heterojunction Nanocomposite

**DOI:** 10.3390/nano12101628

**Published:** 2022-05-10

**Authors:** Hanie Hashtroudi, Aimin Yu, Saulius Juodkazis, Mahnaz Shafiei

**Affiliations:** 1 School of Science, Computing and Engineering Technologies, Swinburne University of Technology, Melbourne, VIC 3122, Australia; aiminyu@swin.edu.au (A.Y.); sjuodkazis@swin.edu.au (S.J.); 2 World Research Hub Initiative (WRHI), School of Materials and Chemical Technology, Tokyo Institute of Technology, 2-12-1, Ookayama, Meguro-Ku, Tokyo 152-8550, Japan

**Keywords:** Conductometric sensors, 2D CeO_2_-Pd-PDA/rGO heterojunction nanocomposite, H_2_ sensing, humidity effect, room and low working temperature, UV radiation effect

## Abstract

A two-dimensional (2D) CeO_2_-Pd-PDA/rGO heterojunction nanocomposite has been synthesised via an environmentally friendly, energy efficient, and facile wet chemical procedure and examined for hydrogen (H_2_) gas sensing application for the first time. The H_2_ gas sensing performance of the developed conductometric sensor has been extensively investigated under different operational conditions, including working temperature up to 200 °C, UV illumination, H_2_ concentrations from 50–6000 ppm, and relative humidity up to 30% RH. The developed ceria-based nanocomposite sensor was functional at a relatively low working temperature (100 °C), and its sensing properties were improved under UV illumination (365 nm). The sensor’s response towards 6000 ppm H_2_ was drastically enhanced in a humid environment (15% RH), from 172% to 416%. Under optimised conditions, this highly sensitive and selective H_2_ sensor enabled the detection of H_2_ molecules down to 50 ppm experimentally. The sensing enhancement mechanisms of the developed sensor were explained in detail. The available 4f electrons and oxygen vacancies on the ceria surface make it a promising material for H_2_ sensing applications. Moreover, based on the material characterisation results, highly reactive oxidant species on the sensor surface formed the electron–hole pairs, facilitated oxygen mobility, and enhanced the H_2_ sensing performance.

## 1. Introduction

Amongst all the clean energy sources, hydrogen (H_2_) is an alternative renewable source used in the carbon-neutral hydrogen technologies that can replace traditional energy sources, such as fossil fuels, to avoid carbon emissions, air pollution, and climate change [1]. Therefore, due to the growing economic demand and energy consumption, H_2_ is now being used in different industries to produce green energy and power for mobile and stationary applications [1,2]. Across the H_2_ supply chain—from generation, transportation, storage, and ultimate use—care must be taken to ensure the safe handling of this volatile fuel [3]. H_2_ gas is lighter than air and tends to accumulate in enclosed spaces from even small leaks. Its broad explosive range (4 to 75%), minimal ignition energy (0.017 mJ), and near colourless flame present significant hazards even under a small-scale leakage [4]. Thus, there is a critical need for the subsequent development of H_2_ sensing and measurement techniques that are accurate, robust, real-time, power efficient, and scalable for deployment over large spatial scales to accommodate critical data for safe, effective, and efficient H_2_ production, storage, and usage [1]. Many semiconducting materials are used for conductometric H_2_ sensing, including graphene-based materials, transition metal dichalcogenides, and metal oxides [5,6]. Many different sensing technologies have been developed for H_2_ sensing [7,8,9] and semiconductor-based H_2_ sensors mainly present high sensitivity, quick response, and good stability based on their physical and electrochemical characteristics [10,11,12,13,14]. Commonly, the exceptional physical, optical, and electrical properties of 2D semiconductors, such as high surface to volume ratios and numerous active sites, lead to promising gas sensing performance (i.e., gas selectivity, excellent response, durability, and quick response and recovery) because of a change in charge density concentration near or on the sensing layer [15,16,17,18].

Moreover, graphene-based H_2_ sensors can be considered suitable devices at low operating temperatures based on their excellent electrochemical stability, low resistance, and high charge carrier mobility [16,19]. However, the absence of a direct bandgap and few dangling bonds on the sensing surface result in slow recovery and poor selectivity [20]. Therefore, hybridising graphene-based materials with metal oxides and/or functionalising them with noble metals can enhance the gas sensing performance by facilitating the dissociation of the gas molecules into the sensing surface and forming a dipole layer [21,22,23,24,25,26,27]. Moreover, based on the literature, UV illumination can considerably improve the recovery and response of the sensors [16,28,29]. Inspired by these studies, in this work, we combined rGO nanosheets (NSs) with Pd nanoparticles (NPs) to improve the electrocatalytic activities using an environmentally friendly and efficient technique to prevent spontaneous agglomeration of rGO NSs [30,31]. The conventional reduction technique limits the anchor sites on rGO for Pd NPs growth in addition to creating toxic and hazardous chemical residues that are damaging to the environment on a large scale [32]. Therefore, we utilised dopamine (DA), which was proved to be a green chemical for GO reduction and functionalisation to reduce the GO NSs and form a self-polymerised polydopamine (PDA) coating to prevent rGO agglomeration due to the loss of oxygen functional groups. The PDA modified rGO NSs were further functionalised with Pd NPs, to form a Pd-PDA/rGO nanocomposite [31,33].

Meanwhile, rare earth metal oxides, including ceria (CeO_2_), have attracted considerable attention in gas sensing applications due to their electrical conductivity, oxygen storage capacity, and oxygen deficiency [34,35,36]. Ceria is an exceedingly versatile material that has recently been used in different applications, including environmental gas monitoring, based on its excellent resistance to chemical corrosion, electrical and optical properties, non-toxicity, thermal stability, safety, and reliability [37]. More importantly, having a number of electrons in its 4f subshell results in significant oxygen mobility, a remarkable oxygen release/storage capability [38], and the unique redox reaction between Ce^4+^ and Ce^3+^ valence states [39], which enhances its gas sensing properties [40].

Although pure ceria has shown poor gas sensing performance because of its wide bandgap (3.1 eV), which requires more energy to excite the electrons to the conduction band, and is functional mainly at high temperatures [41], the modified hybrid structures of ceria have been recently used to detect CO [42], H_2_S [43], NO_2_ [35], H_2_O_2_ [44], C_2_H_5_OH [36], CS_2_ [45], and C_3_H_6_O [46]. However, to the best of the authors’ knowledge, there have been no studies on the H_2_ sensing properties of 2D ceria-based sensors, which is considered to be the initial motivation for this work. In addition, the use of ceria in the presence of noble metals (Pd, Pt, and Au) and hybridising with reduced graphene oxide can form heterojunctions between the material as well as act as a heterogeneous catalyst that enhances either oxidation or hydrogenation reactions [47,48,49]. Especially, Pd NPs have proved to be the best noble metal for H_2_ gas sensing due to their facilitation of the adsorption and diffusion of H_2_ molecules into the sensing layer and the production of PdHx species [50]. Furthermore, Ma et al. [51] reported the H_2_ adsorption capability of ceria, which can play a significant role in the overall efficiency of its H_2_ gas sensing application [40].

Herein, this work reports the development of a photoreactive semiconducting 2D CeO_2_-Pd-PDA/rGO heterojunction nanocomposite for H_2_ gas sensing with enhanced performance at a relatively low working temperature (100 °C). It is worth mentioning that the developed sensor is also functional at room temperature (30 °C). The synthesis technique, material characterisation, and H_2_ sensing mechanisms of the developed 2D CeO_2_-Pd-PDA/rGO heterojunction nanocomposite are fully explained. The experimental results confirm the potential application of 2D CeO_2_-Pd-PDA/rGO heterojunction nanocomposite as a high-performance H_2_ gas sensing device.

## 2. Materials and Methods

### 2.1. Material Synthesis Procedure and Gas Sensor Fabrication

**Figure 1:**
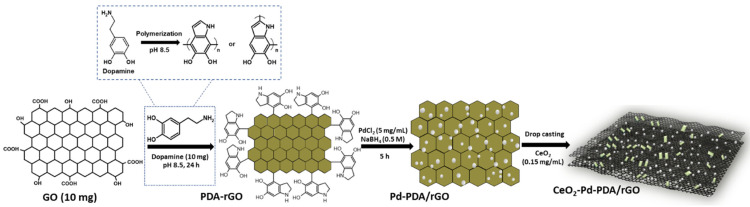
Schematic illustration of the synthesis process of the CeO_2_-Pd-PDA/rGO heterojunction nanocomposite.

Commercially produced graphene oxide (GO) powder was obtained from JCNano Inc. Advanced Materials Supplier, Nanjing, China, and the 2D cerium oxide (CeO_2_) dispersion was purchased from 2D Semiconductors Inc. (Scottsdale, AZ, USA); the concentration was 0.15 mg/mL and the size was less than 100 nm. All the other materials used for the synthesis of CeO_2_-Pd-PDA/rGO heterojunction nanocomposite, (dopamine hydrochloride (DA), palladium (II) chloride (PdCl_2_), and sodium borohydride (NaBH_4_)), were bought from Sigma-Aldrich, NSW, Australia. Figure 1 demonstrates the schematic diagram of an environmentally friendly and facile synthesis technique for the CeO_2_-Pd-PDA/rGO heterojunction nanocomposite [31,33]. At the start, the GO NSs (10 mg) were mixed in 30 mL of Tris buffer (pH 8.5) and sonicated for 1 h. Then, 10 mg of DA was added to the suspension and sonication was continued until DA was fully dissolved. Later, the mixture was put under vigorous shaking conditions for 24 h. The GO was reduced and functionalised with polydopamine (PDA) through these steps via the self-polymerisation process. The DA initiates self-polymerisation to form polydopamine (PDA) in the presence of GO oxygen functional groups in a weak alkaline environment, with the catechol groups undergoing oxidation until producing the quinone groups, resulting in the reduction of GO [31,33].

Afterwards, the synthesised PDA/rGO nanocomposite was sonicated, centrifuged, and washed several times with deionised water (DI). Then, DI water (3 mL) and 1 mL of PdCl_2_ solution (5 mg/mL) were added to 1 mL of PDA/rGO (1 mg/mL) and mixed for 2 h. Next, 1 mL of freshly made NaBH_4_ solution (0.5 M) was added to the mixture and stirred for 5 h. The mixture was then centrifuged and washed 3 times with DI water in order to collect the final product, the Pd-PDA/rGO nanocomposite [33]. Then, 0.5 mL of DI was added to the Pd-PDA/rGO nanocomposite powder to form a homogeneous suspension. Afterwards, 1 µL of Pd-PDA/rGO dispersion (1 mg/mL) was drop-casted onto 10 × 6 mm gold interdigitated electrode fingers with a spacing of ~10 µm as a first layer of the sensor. Finally, 1 µL of CeO_2_ (0.15 mg/mL) dispersion was drop-casted on top of the Pd-PDA/rGO as the second layer to make the CeO_2_-Pd-PDA/rGO heterojunction nanocomposite sensor device.

### 2.2. Material Surface Characterisation

The structural and morphological characteristics of the CeO_2_-Pd-PDA/rGO nanocomposite were analysed using different techniques. The scanning electron microscope (EBL-SEM) (Raith150 Two, Raith Germany Co., Ltd., Dortmund, Germany), the high-resolution transmission electron microscope (HRTEM) JEOL ARM200F’ NeoARM’ (JEOL Ltd., Tokyo, Japan) at 200 kV, and energy-dispersive X-ray spectroscope (EDS) (TESCAN MIRA3 FEG-SEM combined with Thermo Scientific UltraDry EDS, ThermoFisher Scientific, Melbourne, VIC, Australia) were used to investigate the surface morphology and elemental composition of the CeO_2_-Pd-PDA/rGO heterojunction nanocomposite. An X-ray diffractometer (D8-Advanced, Bruker Corporation, Bremen, Germany) with Cu K_α_ and λ = 1.54 Å at 40 kV and 20 mA over the range of 5–95 degrees was applied to determine the crystal phases of the material. Raman spectroscopy (Renishaw plc, Gloucestershire, UK), using 514.5 nm laser excitation, was utilised to measure the Raman spectra of the material.

### 2.3. Gas Sensing Measurements

A gas sensing system [16] was used to examine the gas sensing properties of the conductometric CeO_2_-Pd-PDA/rGO heterojunction nanocomposite-based sensor under different operational conditions. The utilised gas sensing system consists of various parts, including six mass flow controllers (GE50A MFCs) for the regulation of the gas concentrations, temperature and humidity-controlled Linkam stage (T96, Linkam Scientific Instruments Ltd. Tadworth, UK), a built-in heater (LNP96), a Keithly Piccoammeter (model 6487) to periodically measure interval currents throughout the experiments, a humidity generator, and a UV LED (365 nm, M365D1 LED, with 8.9 µW/mm^2^ power-driven with a current of 700 mA), which was installed on top of the Linkam stage. The sensor was tested under different working conditions, including temperatures from RT (30 °C) up to 200 °C, relative humidity from 0 to ~30% RH at 100 °C, and H_2_ concentration from 50 to 6000 ppm. Before each experiment, the sensors were left in pure air for 1 h before the gas exposure. The air/gas mixture flow rate was set at 200 sccm and the target gas exposure time for each experiment was 15 min, followed by two hours of purging for the recovery. For the experiments in the humid condition, the humidity was introduced to the system from the beginning of the experiments and settled for 1 h before gas exposure. The long-term stability and gas selectivity of the sensor were also investigated. The sensor was exposed to 50 ppm of various gases, including hydrogen, nitrogen dioxide, methane, ammonia, and acetone. A bias voltage of 1 V was applied throughout the experiments, and the resistivity change in the sensor was measured upon exposure to the target gas. The response of the sensor (R) was calculated towards the target gas as follows [52,53,54]:

(1) $$R=\left(\frac{R_{g}}{R_{a}}\right)\times 100$$

where *R_a_* is the sensor’s resistivity in air and *R_g_* is the sensor’s resistivity in contact with the target gas. The response and recovery times were calculated when the sensor reached 90% of its response and recovery (back to its initial baseline). Three similar sensors were fabricated for this work, and their gas sensing performance was evaluated. Each experiment was repeated at least three times under the exact operating conditions to validate the reliability and repeatability of the gas sensing process. The gas sensing results of all prepared sensors were comparable.

## 3. Results and Discussion

### 3.1. Material Characterisation Analysis

**Figure 2:**
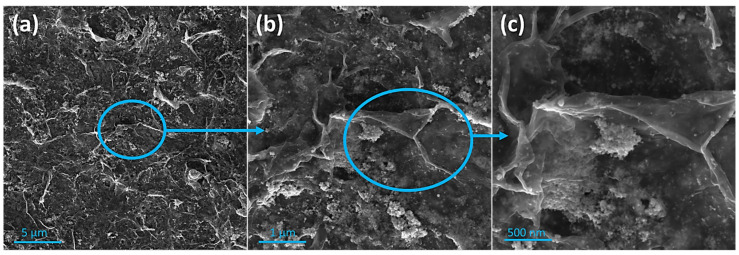
SEM images of the multi-layered porous structure of the CeO_2_-Pd-PDA/rGO heterojunction nanocomposite with wrinkles and open edges at different magnifications. Scale bar: (**a**) 5 µm, (**b**) 1 µm, and (**c**) 500 nm.

The morphology and surface structure of the 2D CeO_2_-Pd-PDA/rGO heterojunction nanocomposite have been analysed by SEM at different magnifications, as shown in Figure 2. The SEM images show the homogeneous distribution of Pd NPs with an average crystalline size of <13 nm and small nanoclusters and the heterogeneous distribution of 2D ceria nanoclusters. When the particle size is small, the surface area increases, resulting in the presence of more active atoms on the surface and consequently more oxygen vacancies accompanied by lattice strain [55]. The lattice parameter is higher for the smaller particle size of CeO_2_ due to the higher concentration of oxygen vacancies and their associated Ce^3+^ for ceria particles [55]. The porous and multi-layered structure of the PDA functionalised rGO can be seen, which confirms the PDA/rGO NSs are interconnected with some folded edges. Moreover, Figure 2b,c indicate that the PDA/rGO NSs are wrinkled and crumpled, showing the good distribution of the Pd NPs and ceria on both sides of the PDA/rGO NSs and the absence of significant agglomerations.

**Figure 3:**
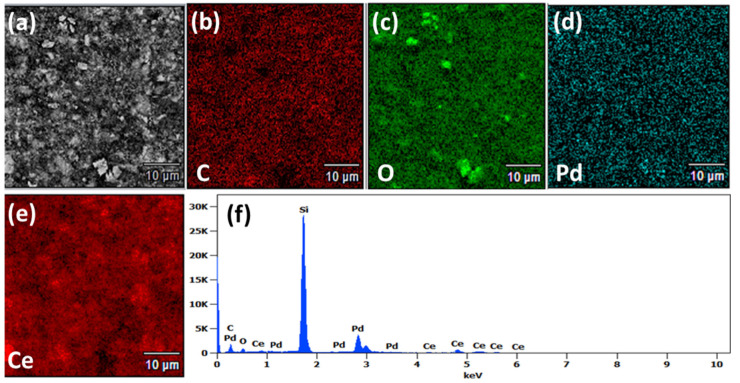
(**a**) SEM image and EDS mapping of (**b**) carbon, (**c**) oxygen, (**d**) palladium, and (**e**) cerium, and (**f**) EDS spectrum of the CeO_2_-Pd-PDA/rGO heterojunction nanocomposite.

Elemental composition and surface coverage of the CeO_2_-Pd-PDA/rGO heterojunction nanocomposite were investigated using EDS and presented in Figure 3. The EDS spectrum (Figure 3f) confirms that the sample (drop-casted on Si substrate) is composed of five elements of carbon, oxygen, palladium, cerium, and Si (substrate). In addition, Figure 3b–e illustrates the scattering of each component on the surface, which confirms the formation of the CeO_2_-Pd-PDA/rGO heterojunction nanocomposite.

**Figure 4:**
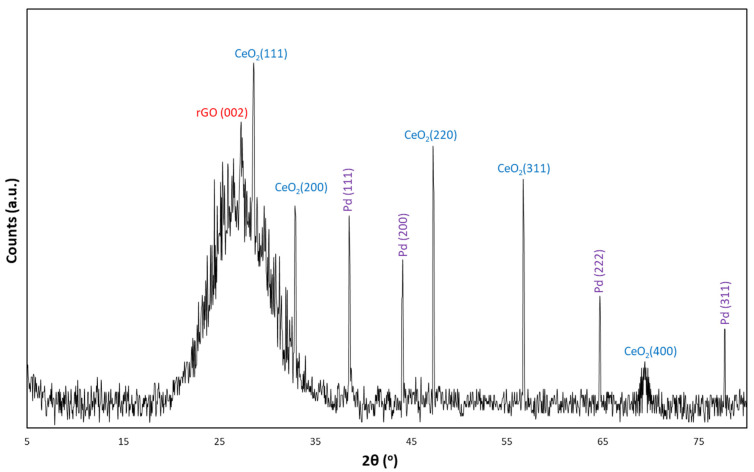
X-ray analysis of CeO_2_-Pd-PDA/rGO heterojunction nanocomposite.

The XRD analysis of the CeO_2_-Pd-PDA/rGO heterojunction nanocomposite is shown in Figure 4 to signify its degree of crystallinity. The XRD profile confirmed a relatively broad peak at 2θ = 27.3° corresponding to the (002) graphitic plane of the partially restacked rGO NSs with the intralayer spacing of 0.326 nm between the rGO NSs, calculated from Bragg’s law (2), as shown below:

(2) $$\lambda =2\mathrm{dsin}\left(\theta \right)$$

where the λ (equals 0.154 nm) is the X-ray beam wavelength, θ is the diffraction angle, and d is the intralayer spacing between the rGO NSs. Subsequently, the Scherrer Equation (3) was used to calculate the graphene layers number in rGO NSs to be less than three layers, as follows:

(3) $$X=\frac{K\lambda }{\beta \cdot \cos\left(\theta \right)}$$

where the X is the number of rGO layers, K is a dimensionless shape factor, λ is the X-ray beam wavelength, β is the line broadening at half the maximum intensity (FWHM), and θ is the Bragg angle.

From the XRD pattern, the high-intensity diffraction peaks observed at 2θ = 28.6, 32.9, 47.3, 56.7, and 69.4 attributed to the (111), (200), (220), (311), and (400) crystalline planes of CeO_2_, respectively, which was a very good fit with the JCPDS data card No. 43-1002 [35]. The results confirm the hexagonal structure of the CeO_2_, which is consistent with the HRTEM results that are presented later in this section. The other sharp peaks at 2θ = 38.55, 44.15, 64.7, and 77.7 are attributed to the (111), (200), (222), and (311) lattice planes of Pd NPs, respectively, which are a good fit to the JCPDS DATA CARD No. 5-681. This result indicates the face-centred cubic (FCC) structure of the Pd NPs inside the rGO NSs [33]. Hydrogen adsorption selectivity of the sensing material is highly correlated to the morphology-dependent oxygen reducibility and oxygen vacancy concentrations [56]. Given that, different morphologies of CeO_2_ possess unique catalytic behaviour corresponding to the specific surface characteristics of each particular lattice plane [57,58]. For instance, the predominant (111) crystal plane is responsible for the selective hydrogenation reaction due to the lowest surface energy and highest oxygen vacancy concentration [59]. In addition, the H_2_ molecules occupying the oxygen vacancies on the sensor’s surface can improve the photocatalytic activity of the material due to the enhanced optical absorption and the bandgap states [48,60].

**Figure 5:**
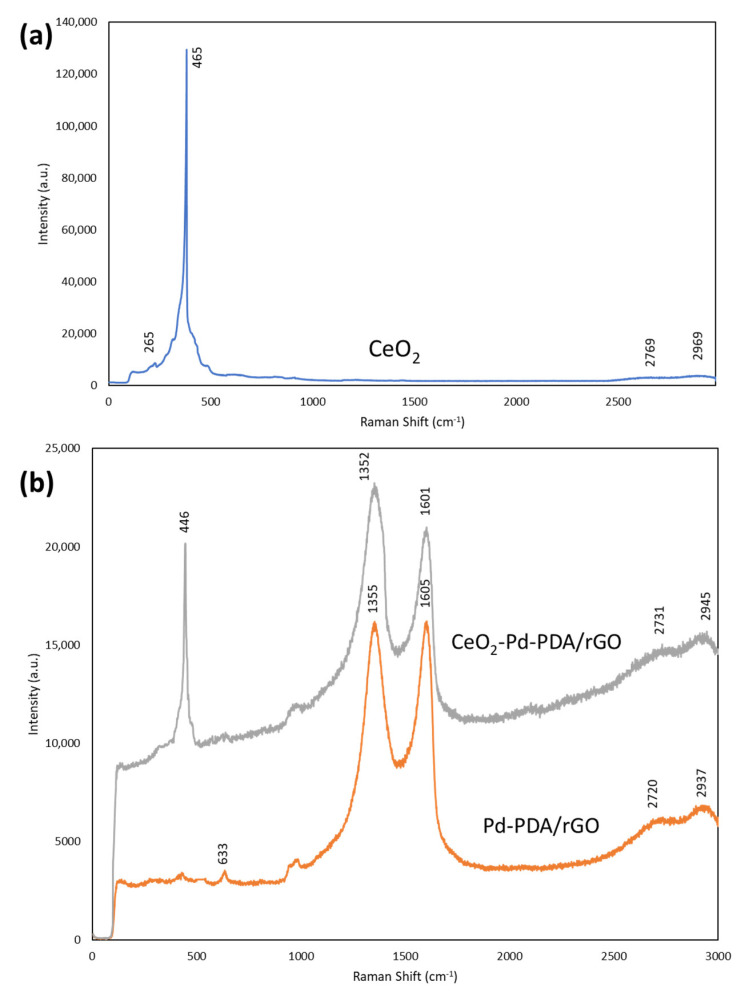
The Raman spectra of the ceria, (**a**) CeO_2_, and (**b**) Pd-PDA/rGO, and CeO_2_-Pd-PDA/rGO nanocomposite.

Raman analysis of the 2D ceria, Pd-PDA/rGO, and CeO_2_-Pd-PDA/rGO heterojunction nanocomposite was carried out using a 514 nm laser spectrometer and presented in Figure 5. Raman spectroscopy provides information about the structural transformation of the material and can detect the formation of defects inside the material. Generally, the oxygen vacancies on the surface of ceria are produced due to the reversible reduction of Ce^4+^ to Ce^3+^, based on the defect chemistry [61]. The concentration of oxygen vacancy is a critical element in the gas sensing characteristics of the ceria. As shown in Figure 5, the Raman spectrum of CeO_2_ shows a sharp peak at ~465 cm^−1^ corresponding to the F_2g_ mode of symmetric Ce-O_8_ stretching vibration in the O_h_ group, which is sensitive to any disorder in the oxygen sublattice [62]. It can be considered a symmetric breathing mode of six oxygen atoms around the central cerium ion. The results confirm the hexagonal shape of the ceria, which is consistent with the TEM results. A small peak at ~265 cm^−1^ can be ascribed to the second-order transverse acoustic mode (2TA). 2D bands can be seen near ~2769 cm^−1^ and ~2969 cm^−1^, which are slightly shifted to higher wavenumbers in comparison with the 2D band of the Pd-PDA/rGO and CeO_2_-Pd-PDA/rGO heterojunction nanocomposite (~2730 cm^−1^ and ~2923 cm^−1^) due to the layer thickness increase [63]. The Raman spectrum of the Pd-PDA/rGO shows two characteristic peaks at ~1355 cm^−1^ and ~1605 cm^−1^ ascribed to the D and G bands, while the D and G bands of CeO_2_-Pd-PDA/rGO are slightly shifted to the lower wavenumbers (~1352 cm^−1^ and 1601 cm^−1^), due to the formation of heterojunction between the ceria and Pd-PDA/rGO, and charge transfer [64]. The presence of D and G bands in two spectra also confirms that the Pd-PDA/rGO NSs structure has been maintained after combining with ceria. The D band is correlated with the density of defects in the sp^2^ material, and the G band corresponds to the active E_2g_ phonon mode of the graphite’s symmetric structure [65,66]. However, the intensity of the D and G bands of the composite material is downshifted due to the electron transfer from the ceria to rGO NSs, confirming the formation of heterojunctions between ceria and Pd-PDA/rGO NSs, which is consistent with the TEM results [64]. The comparison of the Raman spectra of the pure ceria and CeO_2_-Pd-PDA/rGO nanocomposite reveals that the F_2g_ mode (Ce-O_8_ vibration unit) is shifted to the lower wavenumber (~446 cm^−1^) due to the strong interaction between the ceria Pd-PDA/rGO NSs forming the structural defects, such as oxygen vacancies, which facilitate the charge transfer from the ceria to the rGO NSs [67]. The 2D bands are broadened for the nanocomposite, indicating exfoliation of the rGO [67]. In addition, the Raman spectrum of the nanocomposite confirms that the ceria is successfully incorporated into the Pd-PDA/rGO NSs. The defect density on the CeO_2_-Pd-PDA/rGO heterojunction nanocomposite and Pd-PDA/rGO NSs can be calculated by the intensity ratio (*I_D_/I_G_*) of the D band to G band, which is increased by loading the ceria to the Pd-PDA/rGO NSs. The I_D_/I_G_ for Pd-PDA/rGO NSs and CeO_2_-Pd-PDA/rGO nanocomposite were found to be 0.99 and 1.1, respectively, which reflects the higher defect density in the nanocomposite and greater oxygen vacancies on the nanocomposite surface.

**Figure 6:**
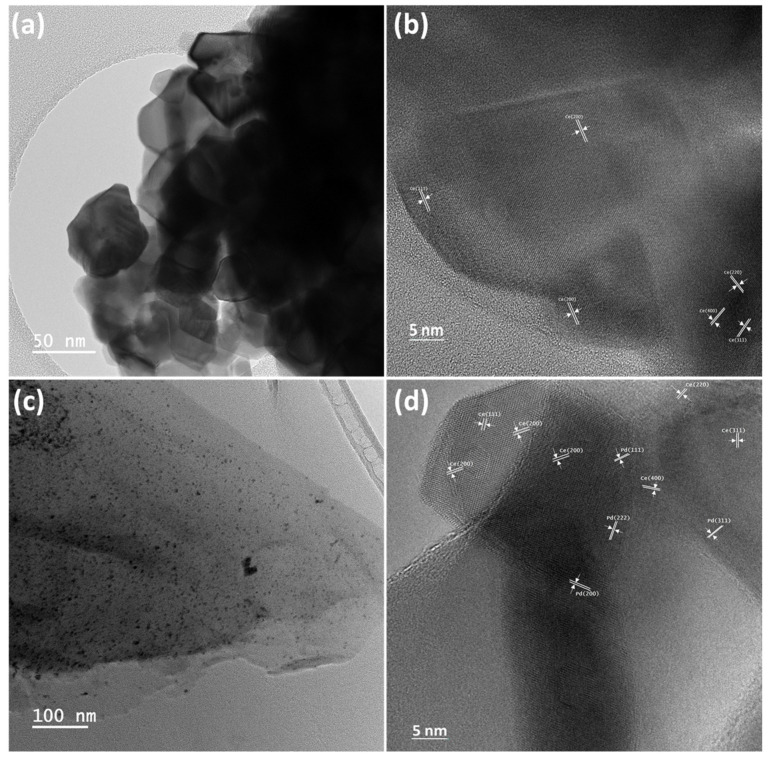
HRTEM images of the CeO_2_ (**a**,**b**) and CeO_2_-Pd-PDA/rGO heterojunction nanocomposite (**c**,**d**) at low (**a**,**c**) and high (**b**,**d**) magnifications that indicate the interplanar spacings of 2D CeO_2_ and Pd NPs.

Crystal structural features and internal morphology of 2D CeO_2_ and CeO_2_-Pd-PDA/rGO heterojunction nanocomposite were investigated by HRTEM, as shown in Figure 6. Figure 6a,b displays the HRTM images of the 2D CeO_2_ in different magnifications, confirming transparency and the 2D structure of the CeO_2_ and the existence of well-defined polycrystalline hexagonal structured CeO_2_ (111), (200), (220), (311), and (400) planes with interplanar spacings of 0.334, 0.272, 0.192, 0.162, and 0.135 nm, respectively. Likewise, Figure 6c,d demonstrates a few layers of transparent and thin film-like structure of the PDA-rGO NSs decorated with the Pd NPs and 2D CeO_2_. It can be seen that the Pd NPs are uniformly dispersed onto PDA/rGO NSs surface with a nearly spherical shape, and CeO_2_ is heterogeneously dispersed into PDA/rGO NSs with some agglomerations. These images prove that the distribution and morphology of the 2D CeO_2_ and Pd NPs into the PDA-rGO NSs are consistent with the SEM images. The lattice fingers of the Pd NPs planes can be seen in Figure 6d. The interplanar spacings of 0.233, 0.205, 0.144, and 0.122 nm are ascribed to the cubic Pd (111), Pd (200), Pd (222), and Pd (311) lattice planes, respectively. According to Bragg’s law (Equation (2)), the XRD pattern of the CeO_2_-Pd-PDA/rGO heterojunction nanocomposite is consistent with the obtained crystal structural features by HRTEM. As shown in Figure 6c,d, the PDA/rGO NSs are relatively thin layers that help to support the Pd NPs and nanoclusters and 2D CeO_2_ to form the CeO_2_-Pd-PDA/rGO heterojunction nanocomposite.

### 3.2. H_2_ Gas Sensing Description

The effect of different operating conditions, including H_2_ concentration (50–6000 ppm), working temperature (30–200 °C), UV (365 nm) illumination, and relative humidity (up to 30% RH) on the H_2_ sensing performance of the 2D CeO_2_-Pd-PDA/rGO heterojunction nanocomposite sensor was investigated, respectively, and reported in the following sections.

#### 3.2.1. Effect of Operating Temperature on the H_2_ Sensing Performance

**Figure 7:**
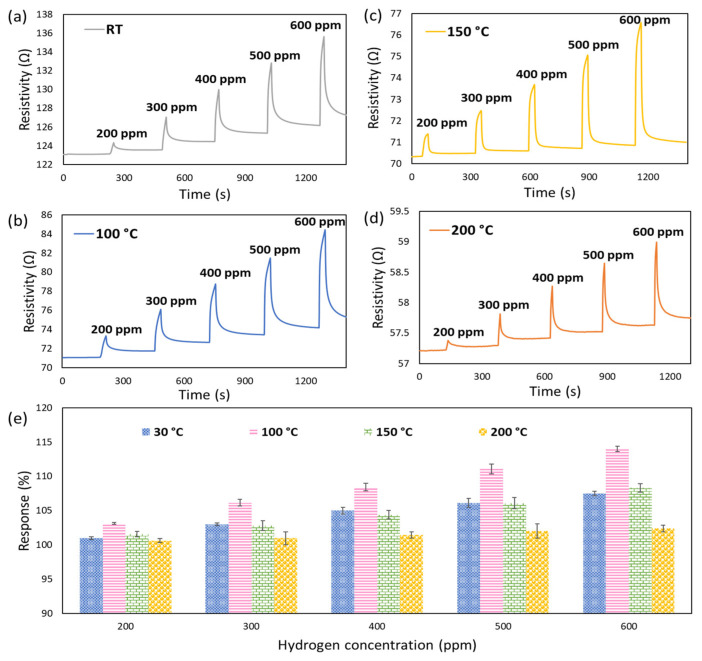
P-type semiconducting gas sensing dynamic responses of CeO_2_-Pd-PDA/rGO sensor towards H_2_ with different concentrations without UV illumination at different working temperatures (**a**) 30 °C, (**b**) 100 °C, (**c**) 150 °C, and (**d**) 200 °C. (**e**) Response values at different operating temperatures and H_2_ concentrations.

The gas sensing properties of semiconductor sensors are greatly dependent on the operating temperature due to the direct relation with their chemo-resistive adsorption-desorption behaviour and, more importantly, the association with power consumption and cost of experiment [68]. Therefore, to optimise the operating temperature as a key element in gas sensing valuation, the dynamic response of the fabricated CeO_2_-Pd-PDA/rGO sensor towards H_2_ with different concentrations (200–600 ppm) was examined at different working temperatures (30 °C up to 200 °C) and 0% relative humidity (% RH) in the dark environment (Figure 7). As shown in Figure 7, the CeO_2_-Pd-PDA/rGO sensor behaves as a p-type semiconductor, where its resistivity increases upon exposure to H_2_ (reducing gas) due to the charge transfer from the target gas molecules to the sensing layer [3]. The electrons move from H_2_, as an electron donor gas, to the valence band of the CeO_2_-Pd-PDA/rGO nanocomposite, which reduces the hole concentration on the surface and, as a result, its conductivity.

The 2D CeO_2_-Pd-PDA/rGO heterojunction nanocomposite-based sensor has shown promising H_2_ sensing responses at low operating temperatures in dark conditions, as shown in Table 1. However, a baseline drift happens due to partly irreversible gas/material chemisorption, indicating that the sensor is not fully recovered to its baseline. This is resolved by introducing UV radiation and changing the charge carrier density on the surface of the CeO_2_-Pd-PDA/rGO by stimulating the electrons from the valence band.

The change in resistivity of the sensor upon exposure to H_2_ gas at different concentrations was measured throughout the experiment. Based on the acceleration of the adsorption/desorption kinetics between the CeO_2_-Pd-PDA/rGO nanocomposite and H_2_ molecules, the sensor’s responses were enhanced by increasing the operating temperature from 30 °C up to 100 °C [6,69,70]. The maximum sensor response at 600 ppm H_2_ was 114% at 100 °C; thus, the 100 °C was chosen as the optimum working temperature. By further increasing the operating temperature above 100 °C, the sensing responses gradually reduced due to the Langmuir effect, where the desorption rate of H_2_ molecules exceeds its adsorption rate on the surface of the CeO_2_-Pd-PDA/rGO nanocomposite [57]. In addition, by increasing the temperature beyond the optimum, the adsorbed H_2_ molecules on the CeO_2_-Pd-PDA/rGO nanocomposite surface might escape before the charge transfer due to the high activation, resulting in a poor response [71].

#### 3.2.2. Effect of UV Radiation on the H_2_ Sensing Performance

**Figure 8:**
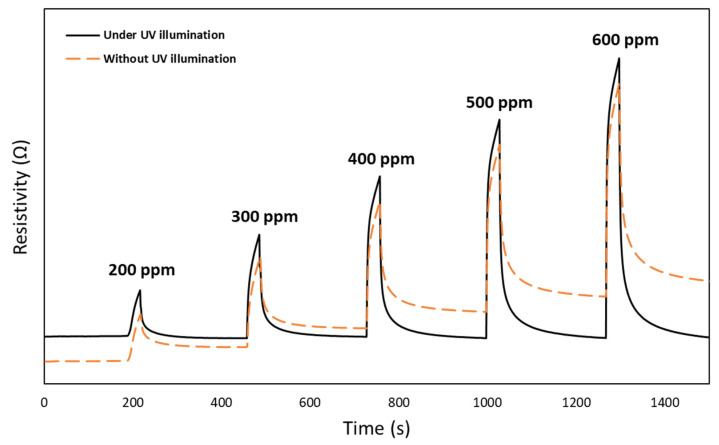
Dynamic response of CeO_2_-Pd-PDA/rGO sensor at 100 °C and 0% RH as a function of H_2_ concentration, without and with UV illumination.

The UV radiance effect on H_2_ sensing performance of the 2D CeO_2_-Pd-PDA/rGO heterojunction nanocomposite-based sensor was investigated at 100 °C and 0% RH as a function of H_2_ concentration. Figure 8 compares the dynamic response of the sensor upon exposure to H_2_ with different concentrations (200–600 ppm) at 100 °C and 0% RH without and with UV (365 nm) illumination. The resistivity change depends on the adsorption/desorption rates of the H_2_ molecules onto the CeO_2_-Pd-PDA/rGO sensing layer, which is correlated to the H_2_ concentrations [72]. By increasing the gas concentration, the number of gas molecules interacting with the surface of the sensing layer rises until the adsorption and desorption reach a balanced state [73]. Figure 8 compares the dynamic response of the sensor in the dark and under UV illumination, which indicates that the sensor was not fully recovered to its original baseline in the dark. However, the sensor is fully recovered by introducing the UV, and no drift has happened in the baseline. These results reveal that the interaction between the H_2_ molecules and the CeO_2_-Pd-PDA/rGO sensing layer under UV radiation is fully reversible [12,74,75].

The UV photoexcitation weakens the bonding forces between the H_2_ molecules and CeO_2_-Pd-PDA/rGO nanocomposite sensing layer throughout the physisorption, leading to reversible adsorption/desorption processes [74,76,77]. Moreover, UV illumination stimulates the electrons from the valence band of the CeO_2_-Pd-PDA/rGO surface [78]. It changes the charge carrier density on its surface and consequently accelerates the adsorption of H_2_ molecules by these photogenerated electrons from the pre-adsorbed ambient oxygen species [78]. As shown, the oxygen vacancies play a significant role in the photocatalytic activities and adsorption behaviour in semiconducting metal oxides, including CeO_2_, due to the creation of isolated levels below the conduction band [79]. In addition, ceria is a photocatalyst with a 4f electron configuration, which improves its adsorption capacity, especially under UV illumination. When exposed to H_2_ as a reducing agent, the electrons transfer into the Ce_4f_ orbitals and consequently to oxygen, forming superoxide anion radicals [40]. In other words, under UV illumination, highly reactive oxidant species including °OH, HO_2_°, and O_2_°^−^ can be produced on the CeO_2_-Pd-PDA/rGO heterojunction nanocomposite surface, resulting in electron–hole pair formation, i.e., where the electrons are excited from the valence band to the conduction band, leaving holes in the valence band [80]. These photoproduced electron–hole pairs migrate to the surface, giving rise to the reduction reaction of the electron acceptor (O_2_) to active oxygen (°O_2_^−^) and the holes oxidise H_2_ (the electron donor), forming hydroxyl radicals and/or H_2_O. The active °O_2_^−^ and OH° facilitate the oxidisation of the adsorbed H_2_ molecules, improving its H_2_ sensing performance [81].

The response values of the CeO_2_-Pd-PDA/rGO sensor without and with UV illumination at 100 °C and 0% RH were analysed for each experiment at different H_2_ concentrations (200–600 ppm) and reported in Table 2.

Table 2 confirms an enhancement in the H_2_ sensing responses of the sensor under UV radiation, which can be attributed to the reduction in the adsorption energy barrier between the H_2_ molecules and the photo-induced sensing layer [82]. In addition, the wide bandgap of ceria (3.1 eV) needs UV illumination to enhance its photocatalytic reaction, resulting in increased numbers of oxygen vacancies in the CeO_2_ lattice and Ce^3+^ ions on the surface [83]. Therefore, increasing the number of H_2_ molecules occupying the oxygen vacancies on the sensor’s surface improves the sensor’s gas sensing activity based on the UV light effect of shifting the ceria’s bandgap and accelerating the adsorption rate [48,60]. However, the gas sensing mechanism of the CeO_2_-Pd-PDA/rGO heterojunction nanocomposite based-sensor is complicated and cannot be generalised due to various physical and chemical adsorption/desorption interactions, such as π-π interactions, van der Waals forces, oxygen molecule adsorption/desorption, formation of different chemical bonds, and charge transfer between the H_2_ molecules and the sensing layer [70,84].

#### 3.2.3. Relative Humidity Effect on the H_2_ Sensing Performance

The above discussed H_2_ sensing parameters of the CeO_2_-Pd-PDA/rGO nanocomposite sensor was investigated under dry ambient conditions. One of the critical environmental elements affecting the H_2_ sensing parameters is relative humidity (% RH) [85]. Water molecules in humid environments contribute to the adsorption/desorption mechanism, influencing physisorption and chemisorption processes [76,86]. Therefore, we studied the effect of the humid environment, changing from a dry ambient up to 30% RH, on the CeO_2_-Pd-PDA/rGO sensing parameters at different H_2_ concentrations and 100 °C. All experiments were performed under UV (365 nm) illumination.

Table 3 presents all the calculated H_2_ sensing response magnitudes of the CeO_2_-Pd-PDA/rGO sensor towards H_2_ concentrations of 4000 and 6000 ppm at different % RH. As indicated in Table 3, the sensor’s responses are improved by raising the H_2_ concentration from 4000 to 6000 ppm. The sensing mechanism of the p-type CeO_2_-Pd-PDA/rGO heterojunction conductometric sensor is based on ion-sorption of H_2_ molecules, followed by a charge transfer directly between the H_2_ molecules and heterostructure-based CeO_2_-Pd-PDA/rGO that produces a variation in the Fermi level (i.e., change in the electrical conductivity). The presence of more H_2_ molecules on the surface facilitates the electron transfer from the H_2_, as an electron donor gas, to the valence band of the CeO_2_-Pd-PDA/rGO nanocomposite, decreasing the hole concentration on the surface and improving the response.

It can also be seen that the response magnitude increases by enriching the % RH up to 15%, which may be due to the participation of water molecules in physical and chemical adsorption/desorption reactions [87]. The water molecules act as electron donors, so by increasing the humidity percentage, the number of adsorbed water molecules increases on the surface, resulting in a narrowing of the depletion region and an increase in the sensor’s resistance [87,88]. This reaction leads to an enhancement in gas sensing response for the p-type CeO_2_-Pd-PDA/rGO sensor.

Moreover, carbonyl and hydroxyl functional groups on the CeO_2_-Pd-PDA/rGO nanocomposite surface facilitate its interaction with water molecules, consequently changing the conductivity [89]. On the other hand, the surface-adsorbed active oxygen species play a significant role in the H_2_ sensing performance. The interaction between the oxygen species on the CeO_2_-Pd-PDA/rGO surface and adsorbed H_2_ molecules can form the hydroxyl active sites, improving the response [90].

Table 3 also reveals that the response magnitude declines by further elevating the humidity up to 30%. With increasing the % RH beyond 15%, the excess water molecules on the surface cause widening of the bandgap by breaking the material sublattice and symmetry (especially the rGO), which leads to a change in conductivity and a reduction in the response [91]. Moreover, in an environment with a high humidity level, the water molecules cannot be readily adsorbed on the surface of the sensing layer because the size of water molecules is greater than H_2_ molecules [85]. Therefore, the response of the sensor decreases.

Table 4 compares the calculated H_2_ sensing parameters of the CeO_2_-Pd-PDA/rGO sensor, including response magnitude and response and recovery times towards H_2_ with various concentrations at 0 and 15% RH.

It can be seen that by raising the H_2_ concentration, the response of the sensor increases, which can be attributed to the increased number of H_2_ molecules interacting with the sensor, enhancing its adsorption rate on the surface of the sensing layer. This continues until the sensor becomes saturated and the adsorption and desorption rate reach a balance.

As shown in Table 4, the maximum response of the CeO_2_-Pd-PDA/rGO sensor was 172% in dry ambient conditions towards 6000 ppm H_2_ at 100 °C under UV radiation. However, the sensor response drastically improved, to 416%, by raising the humidity to 15% RH under the same operational conditions. This can be attributed to the competition between the H_2_ and water molecules to be adsorbed on the sensing layer’s surface [75,92]. The H_2_O molecules participate in the adsorption process in the humid environment, influencing the chemisorption and physisorption sensing mechanisms [6,76,92]. In addition, the high response of the sensor and its sensing improvement in a humid environment can be due to the hydroxylation reaction of the water molecules and the reaction between the hydrogen and oxygen ions ($O_{\left(ads\right)}^{2-}$ and $O_{\left(ads\right)}^{-}$) on the sensing layer surface [93].

Table 4 also reveals that response and recovery times of the sensor were improved by introducing 15% RH. Quick response and recovery of 70 s and 180 s were observed when the sensor was exposed to 6000 ppm H_2_ at 100 °C and 15% RH under UV due to the high permeability of the CeO_2_-Pd-PDA/rGO sensor to water molecules.

**Figure 9:**
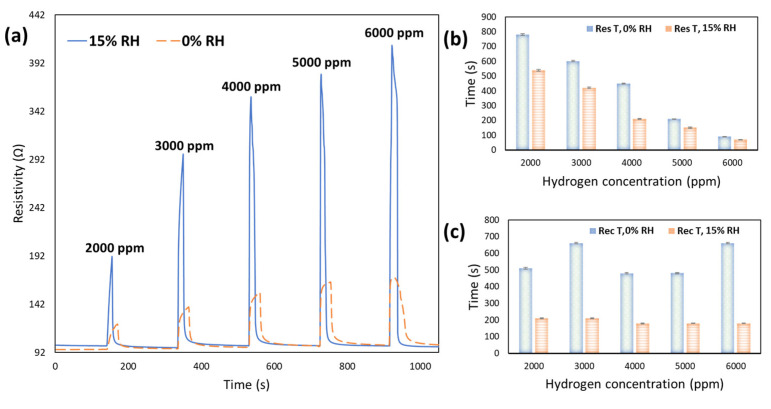
(**a**) The UV excited dynamic responses of CeO_2_-Pd-PDA/rGO sensor at 100 °C and various H_2_ concentrations (2000–6000 ppm) in dry ambient conditions and under 15% relative humidity; (**b**) response times of the sensor at 0 and 15% RH as a function of H_2_ concentration; (**c**) recovery times of the sensor at 0 and 15% RH and different H_2_ concentrations.

Figure 9a demonstrates the dynamic response of the sensor at 0% and 15% RH at H_2_ with different concentrations and 100 °C. Figure 9b,c compares the response and recovery times trends while increasing the humidity and H_2_ concentrations.

Comparing these dynamic responses reveals that a slight drift happens when the sensor is functioning at 0% RH, showing that the sensor doesn’t fully recover, which affects the response. It could be because of some irreversible chemisorption reactions due to the high number of H_2_ molecules interacting with the sensing surface at high H_2_ concentrations. However, when the sensor operated at 15% RH, it is fully recovered to its original baseline after each H_2_ exposure as a result of the presence of H_2_O molecules.

In a humid environment, the response is improved because of the presence of hydroxyl active sites (as electron donors) on the surface, which enhances the electric charge density by forming the hydronium cations from the ionised H_2_O molecules as in [76,86]:

2H_2_O ↔ OH^−^ + H_3_O^+^ (4)

In addition, it is observed that by increasing the H_2_ concentration from 2000 to 6000 ppm, the response and recovery become quicker due to an increase in the surface coverage rate, accelerating the adsorption/desorption process of the H_2_ molecules [70]. Moreover, the sensor reaches its saturation level quicker when exposed to higher H_2_ concentrations. Another critical element affecting the fast response and recovery is the formation of the covalent bonds on the CeO_2_-Pd-PDA/rGO sensing layer because of the participation of the hydroxyl and carbonyl groups in the H_2_ physisorption and chemisorption reactions and also their interactions with water molecules on the surface [94]. The H_2_O molecules are known as electron donors, facilitating the ionisation of the OH and COOH functional groups on the sensing layer surface to generate a concentration gradient of protons [22]. This gradient induces the diffusion of the protons to the CeO_2_-Pd-PDA/rGO nanocomposite, carrying the voltage and current in the external circuit that accelerates the response [95,96].

#### 3.2.4. Gas Selectivity and Sensor Stability

**Figure 10:**
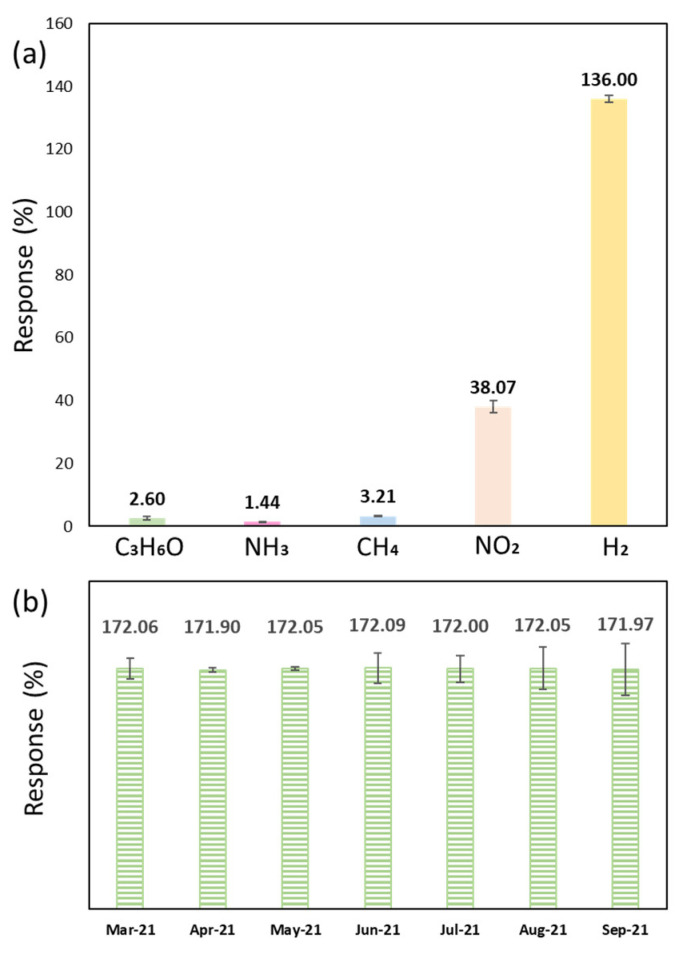
(**a**) Gas selectivity of the CeO_2_-Pd-PDA/rGO sensor at 100 °C and 0% RH towards different gases at 50 ppm; (**b**) long-term sensor stability towards 6000 ppm H_2_ at 100 °C and 0% RH over seven months.

Gas selectivity of the CeO_2_-Pd-PDA/rGO sensor was examined at 0% RH and optimum operating temperature of 100 °C and displayed in Figure 10a. The sensor was exposed to 50 ppm of H_2_, nitrogen dioxide (NO_2_), acetone (C_3_H_6_O), ammonia (NH_3_), and methane (CH_4_) for 30 min. It can be seen that the sensor was highly responsive to H_2_ in comparison with the other gases. The sensor showed a 136% response to H_2_ while indicating a low response towards NH_3_ (1.4%), C_3_H_6_O (2.6%), and CH_4_ (3.2%). Although the sensor showed a 38% response to NO_2_, it was more selective and responsive to H_2_.

The p-type CeO_2_-Pd-PDA/rGO nanocomposite sensing mechanism is mainly based on the charge transfer from the H_2_ gas to the sensing material along with the adsorption/desorption interactions of the H_2_ molecules with the oxygen active sites on the surface [34,97,98]. The free electrons on the CeO_2_-Pd-PDA/rGO nanocomposite surface interact with oxygen molecules in the air to produce reactive oxygen species (O^2−^, O_2_^−^, and O^−^) ions. These reactive ions interact with the H_2_ molecules as an electron donor gas, improving the electron charge transfer to the sensing layer, lowering the hole concentration, and rising resistivity [3,34,99]. As mentioned before, the H_2_ adsorption selectivity of the CeO_2_-Pd-PDA/rGO is highly correlated to its morphology due to oxygen vacancy concentrations [56]. Therefore, the presence of the CeO_2_ (111) facet facilitates the selective hydrogenation reaction based on its lowest surface energy and highest oxygen vacancy concentration [59]. The CeO_2_-Pd-PDA/rGO sensor was repeatedly tested over seven months at 100 °C and 0% RH towards 6000 ppm H_2_. Figure 10b indicates comparable responses of the sensor over time, confirming its long-term stability and lack of significant degradation.

## 4. Discussion and Conclusions

An environmentally friendly wet chemistry procedure was used to synthesise the CeO_2_-Pd-PDA/rGO heterojunction nanocomposite, and its H_2_ gas sensing behaviour was thoroughly investigated under different working conditions, including temperature up to 200 °C, H_2_ concentration from 50–6000 ppm, UV radiation, and relative humidity up to 30% RH. The fabricated p-type sensor performed rapidly with a maximum response of 172% towards 6000 ppm H_2_ at the optimum operating temperature of 100 °C in dry ambient conditions, due to the enhanced number of H_2_ molecules interacting with the sensing material, accelerating the adsorption/desorption rate on the surface until a balanced state is reached. The heterostructured CeO_2_-Pd-PDA/rGO nanocomposite also showed an excellent response, of 416%, to 6000 ppm H_2_ at 15% RH and 100 °C, with high selectivity compared to other gases. This could be attributed to the high volume to surface area ratio of the hybrid sensing material along with the presence of defects on the surface related to oxygen vacancies, as well as the heterojunction interface between the rGO, Pd NPS, and 2D ceria, which leads to additional reaction sites [100]. The surface physical characteristics of the sensor confirmed a heterogeneous distribution of the 2D CeO_2_ and Pd NPs onto the PDA/rGO nanosheets. In addition, the multi-layered porous structure of the CeO_2_-Pd-PDA/rGO nanocomposite improved its H_2_ gas sensing characteristics. The sensor efficiently exhibited a high response of 136% at a low concentration (50 ppm) of H_2_.

The gas sensing mechanisms of hybrid nanocomposites are still under debate. Many surface characteristics can influence the gas sensing performance, including the grain size, crystal orientation, surface thickness, and lateral dimension, which require further systematic studies on the hybridisation impact on the material’s physical, chemical, and electrical characteristics, consequently improving the sensing properties.

However, for p-type conductometric gas sensors in contact with reducing gas, the primary sensing mechanism is based on molecular adsorption and chemisorption of the oxygen species on the surface followed by a charge transfer from the reducing gas molecules to the heterostructure nanocomposite leading to a change in the Fermi level and electrical resistance [24]. The surface morphology and particle size of the materials also influence the H_2_ sensing properties. As mentioned before, the grain size highly affects the activity of the sensing layer and the oxygen vacancies on the surface. When the particle size decreases, the surface area and the available active atoms on the surface increase, i.e., more oxygen vacancies accompanied by lattice strain [55]. When the CeO_2_-Pd-PDA/rGO nanocomposite is exposed to H_2_ as a reducing gas, Ce^3+^ is generated through the reduction of Ce^4+^ by the electron left behind due to oxygen vacancies. Therefore, as the concentration of oxygen vacancies increases, the CeO_2_ lattice parameter rises [55]. In addition, hybridising the Pd decorated rGO NSs with ceria in the form of the p–n heterojunction nanocomposite has shown a promising influence on the H_2_ sensing performance at a low operating temperature (100 °C) with enhanced sensing properties compared to a Pd-rGO sensor [16] and ceria sensor [39].

Table 5 compares the H_2_ sensing parameters of the present work with different graphene-based materials functionalised by noble metals and/or metal oxides and pure CeO_2_. The adsorbed oxygen species on the sensing surface react with H_2_ and form H_2_O molecules, as shown below:

H_2_ → 2H° (5)

2 H° (ads) + O^−^ (ads) → H_2_O + e^−^ (6)

Since ceria is an n-type semiconductor with a bandgap of 3.1 eV, the presence of UV light reduces its bandgap and activates its photocatalytic reactions. On the other hand, when ceria is exposed to a reducing gas such as H_2_, it releases oxygen, forming suboxides as follows [40]:

CeO_2_ + xH_2_ ↔ CeO_2−x_ + xH_2_O + xV (7)

where V is an oxygen vacancy. Doping the ceria with noble metals such as Pd can lower the reduction temperature, while generally, the reduction requires high temperatures [101]. Doping the ceria also introduces a lattice strain that weakens Ce-O bonds, which reduces oxygen vacancy formation energy and, consequently, alters oxygen ion diffusion [101]. In addition, a further beneficial effect is the formation of Pd-O-Ce bonds in the interface, which are proposed as the active sites on Pd/CeO_2_ where chemisorbed H_2_ reacts with lattice oxygen [102,103]. In this process, at the optimum temperature, the PdO/Pd phase transforms to Pd-O-Ce, allowing the Pd NPs to adsorb H_2_ at low temperatures [103]. Consequently, there is a synergic effect between ceria and Pd, which helps the reducibility of the Pd at a low temperature due to the formation of active interfacial sites [37]. This synergic effect also benefits the material’s durability due to the Pd NPs anchoring to the support with Pd-O-Ce bonds [103].

On the other hand, when the CeO_2_(111) is exposed to H_2_ gas, the H atoms sit on top of the O atoms on the surface, forming hydroxyl O-H bonds [104]. The adsorption of H atoms on the ceria surface leads to the reduction of Ce ions based on the electron transfer from the H 1s to Ce 4F orbital [104]. Therefore, the reduced Ce ions can trap the H_2_ and increase the response. Hybridising CeO_2_ with rGO also affected the sensing performance via the formation of a p–n heterojunction between the rGO and CeO_2_ and also the appearance of C-O bonding at the interface between rGO and ceria. In summary, the CeO_2_-Pd-PDA/rGO heterojunction nanocomposite can produce highly reactive oxidant species including °OH, HO_2_°, and O_2_°^−^ under UV illumination, where the electrons are excited from the valence band to the conduction band, leaving holes in the valence band. This results in the formation of photoproduced electron–hole pairs, which can migrate to the surface, giving rise to the reduction reaction by adsorbed H_2_ molecules on its surface.

The H_2_ adsorption selectivity of the sensing material is highly correlated to the morphology-dependent oxygen reducibility and oxygen vacancy concentrations [56]. Given that, different morphologies of CeO_2_ possess unique catalytic behaviour corresponding to the specific surface characteristics of each particular lattice plane [57,58]. For instance, the predominant (111) crystal plane is responsible for the selective hydrogenation reaction due to the lowest surface energy and highest oxygen vacancy concentration [59]. In addition, the H_2_ molecules occupying the oxygen vacancies on the sensor’s surface can improve the photocatalytic activity of the material due to the enhanced optical adsorption and the bandgap states [48,60].

More importantly, one of the critical parameters influencing the H_2_ sensing performance is the adsorption of atmospheric H_2_O molecules as a significant source of interference [17,26]. According to the literature, the effect of humidity on the gas sensing parameters is a complex challenge for most resistive H_2_ sensors [17,105,106]. However, fabricating H_2_ sensors capable of detecting H_2_ in a humid environment is necessary for real-world operating conditions [107]. Therefore, the effect of the % RH on the H_2_ sensing performance of the 2D CeO_2_-Pd-PDA/rGO heterojunction nanocomposite-based sensor was investigated in this study. The response of the sensor was drastically enhanced from 172% to 416% by increasing the humidity to 15% at 6000 ppm H_2_ and 100 °C under UV light. The response and recovery times also improved from 90 s to 70 s and 660 s to 180 s, respectively. This result can be ascribed to increasing the charge carriers on the sensing layer surface by enriching the % RH because of the adsorbed ionised water molecules. Water is known as a reducing agent and transfers electrons to the sensing layer. In a humid environment, the following reactions can happen:

OH^−^ + h^+^_(valent band)_ ↔ °OH (8)

H_2_O + h^+^_(valent band)_ ↔ °OH + H^+^ (9)

OH^−^ + h^+^_(valent band)_ ↔ °OH (10)

O_2_ + e^−^_(conduction band)_ ↔ HO_2_° (11)

While exposed to H_2_O molecules, the transition of electrons into the Ce_4f_ orbitals and ambient oxygen develops highly reactive oxidant species including °OH, HO_2_°, and O_2_°^−^ on the CeO_2_-Pd-PDA/rGO surface, forming the electron–hole pairs and improving the sensing performance of the material [80,84].

**Table 5:** Summarising H_2_ sensing parameters of different graphene-based materials functionalised by noble metals and/or metal oxides and a pure CeO_2_ compared with this work.

Base Material	Hybrid Material	Synthesis Method	H_2_ Conc. (ppm)	Temp. (°C)	RH (%)	Response (%)	Response Time (s)	Recovery Time (s)
rGO [108]	WO_3_- Pd NPs	Hydrothermal	100	RT	-	38	52	155
rGO [109]	SnO_2_- Pd NPs	Microwave synthesis	10,000	RT	-	3	7	6
rGO [110]	NiO	Freeze drying	10,000	50	-	0.64	28	142
Graphene [111]	Pd NPs-SiO_2_	Thermal CVD	500	RT	-	4.1	213	600
rGO [16]	Pd NPs	Wet chemistry	5000	100	10	18.2	170	1440
rGO [112]	Pd-Pt	Hummers’ method, Hydrothermal	8000	25	-	0.52	300	600
rGO [109]	Pt-SnO_2_	Hummers’ method, Hydrothermal	5000	RT	-	3	3	2
CeO_2_ [39]	-	Wet chemistry	100	400	-	2	420	660
CeO_2_ [39]	Pd NPs	Wet chemistry	100	350	-	19	60	360
PDA/rGO (this work)	Pd NPs- 2D CeO_2_	Wet chemistry	6000	100	15	416	70	180

As shown in Table 5, the H_2_ gas sensing parameters of the developed 2D CeO_2_-Pd-PDA/rGO nanocomposite-based sensor are more promising in comparison with the reviewed literature, including pure ceria, Pd-rGO, and metal oxide-doped rGO sensors. Based on the experimental results, reducing the GO by adding the DA, as an environmentally friendly chemical, considerably improved its reduction process, affecting the sensing performance. Moreover, hybridising the PDA/rGO with Pd NPs and 2D CeO_2_ suggestively enhanced the H_2_ sensing parameters [23]. Last, but not least, the fabricated 2D CeO_2_-Pd-PDA/rGO heterojunction nanocomposite was highly selective to H_2_ and displayed acceptable durability over seven months, as well as a very high response and quick response and recovery, and ability to function at low operating temperature.

## Figures and Tables

**Table 1:** Hydrogen sensing response of CeO_2_-Pd-PDA/rGO heterojunction nanocomposite at different operating temperatures and H_2_ concentrations in the dark.

H_2_ Concentration (ppm)	Response (%)			
	30 °C	100 °C	150 °C	200 °C
200	101	103	102	100
300	103	106	103	101
400	104	108	104	102
500	106	111	106	102
600	108	114	108	102

**Table 2:** H_2_ sensing response of the CeO_2_-Pd-PDA/rGO heterojunction nanocomposite sensor towards H_2_ with different concentrations without and with UV illumination at 100 °C and 0% RH.

H2 Concentration (ppm)	200	300	400	500	600
Response (%), without UV	103	106	108	111	114
Response (%), with UV	103	107	111	115	119

**Table 3:** H_2_ sensing response of the CeO_2_-Pd-PDA/rGO sensor towards 4000 and 6000 ppm H_2_ at various relative humidity and 100 °C under UV (365 nm) illumination.

H_2_ Conc (ppm)	Response (%)					
	0% RH	10% RH	15% RH	20% RH	25% RH	30% RH
4000	160	217	361	225	192	220
6000	172	349	416	373	333	257

**Table 4:** Sensing parameters towards H_2_ different concentrations at 100 °C, 0% RH, and 15% RH under UV (365 nm) illumination.

H_2_ Conc (ppm)	Response (%)		Response Time (s)		Recovery Time (s)	
	0% RH	15% RH	0% RH	15% RH	0% RH	15% RH
2000	127.2	193.5	780	540	510	210
3000	145.6	307	600	420	660	210
4000	160	361	450	210	480	180
5000	168	386	210	150	480	180
6000	172	416	90	70	660	180

## Data Availability

Not applicable.
